# Long-Term Survival in Patients with or without Implantable Cardioverter Defibrillator after Transcatheter Aortic Valve Implantation

**DOI:** 10.3390/jcm10132929

**Published:** 2021-06-30

**Authors:** Ulrich Fischer-Rasokat, Matthias Renker, Christoph Liebetrau, Maren Weferling, Andreas Rolf, Andreas Hain, Johannes Sperzel, Yeong-Hoon Choi, Christian W. Hamm, Won-Keun Kim

**Affiliations:** 1 Department of Cardiology and Cardiac Surgery, Kerckhoff Heart Center, Benekestr. 2-8, 61231 Bad Nauheim, Germany; m.renker@kerckhoff-klinik.de (M.R.); Christoph.liebetrau@googlemail.com (C.L.); m.weferling@kerckhoff-klink.de (M.W.); a.rolf@kerckhoff-klinik.de (A.R.); a.hain@kerckhoff-klinik.de (A.H.); j.sperzel@kerckhoff-klinik.de (J.S.); y.choi@kerckhoff-klink.de (Y.-H.C.); c.hamm@kerckhoff-klinik.de (C.W.H.); w.kim@kerckhoff-klink.de (W.-K.K.); 2 German Centre for Cardiovascular Research (DZHK), Partner Site RheinMain, 61231 Bad Nauheim, Germany; 3 Cardioangiological Center Bethanien (CCB), Im Prüfling 23, 60389 Frankfurt, Germany; 4 Medical Clinic I (Cardiology and Angiology), University Hospital of Giessen, Klinikstr. 33, 35392 Giessen, Germany

**Keywords:** aortic stenosis, implantable cardioverter defibrillator, survival, TAVI

## Abstract

Patients with symptomatic aortic stenosis (AS) can have concomitant systolic heart failure (HF) that persists even after correction of afterload by transcatheter aortic valve implantation (TAVI). These patients qualify as potential candidates for prophylactic therapy with an implantable cardioverter defibrillator (ICD). We compared survival between patients with or without an ICD after successful TAVI. This retrospective study analyzed Kaplan-Meier survival data during a follow-up period of three years in two populations: (a) patients with a left ventricular ejection fraction (LVEF) ≤ 35% before TAVI (overall population); (b) patients with additionally documented LVEF ≤ 35% 3 months after TAVI (persistent LV dysfunction subpopulation). In the overall population, 53 patients with and 193 patients without an ICD had similar baseline characteristics and procedural success rates, and HF medication at discharge was comparable. Three-year mortality rates were 26.4% for patients with an ICD and 24.4% for patients without an ICD (*p* = 0.758). Cardiovascular death rates were similar between groups (*p* = 0.914), and deaths were most often attributed to worsening of HF. Survival rates in patients with persistent LV dysfunction with an ICD (*n* = 24) or without an ICD (*n* = 59) were similar between groups (*p* = 0.872), with cardiovascular deaths mostly qualified as worsening HF and none as sudden cardiac death. Patients of the overall study population with biventricular pacing devices showed only a tendency to have better outcomes (*p* = 0.298). ICD therapy in elderly patients with AS and LV dysfunction undergoing TAVI did not demonstrate a survival benefit during a 3-year follow-up period.

## 1. Introduction

Implantable cardioverter defibrillator (ICD) therapy is recommended for primary prophylaxis in patients with symptomatic heart failure (HF) and a left ventricular (LV) ejection fraction (EF) ≤ 35% in addition to optimal medical therapy [1]. Certain patients undergoing transcatheter aortic valve implantation (TAVI) for severe, symptomatic aortic stenosis (AS) may fulfill these criteria. Supporting arguments for the indication of ICD therapy in these patients include clinical and laboratory characteristics like those of HF patients [2] together with a high rate of prior cardiac decompensation, [3,4] persistent structural and functional impairment of the LV after correction of AS, [5,6] and a post-interventional course comparable to that of typical HF patients characterized by a high rate of hospitalization and poor survival [3,4,7]. Such patients are diagnosed with concomitant HF and should be treated by guideline-directed therapies. On the other hand, potential post-interventional recovery of the LVEF, advanced age and limited life expectancy, suboptimal medical therapy, socioeconomic reasons, and the complete lack of data on potential benefits of ICD in these patients have led to a reluctance to implant ICDs. This study analyzed the association of ICD therapy with survival after TAVI in patients with a pre-interventional LVEF ≤ 35%.

## 2. Methods

### 2.1. Study Design, Participants, and Setting

This is an observational study employing a retrospective analysis. Patients undergoing transfemoral TAVI for symptomatic, severe AS (aortic valve area index < 0.6 cm^2^/m^2^ body surface area or transvalvular mean pressure gradient ≥ 40 mmHg) at a single, high-volume center were included consecutively in an observational registry from January 2011 until December 2020. Only patients discharged from hospital after successful TAVI (exclusion for intraprocedural conversion to open surgery, exclusion for in-hospital death) with documented medical therapy at discharge were included. Two patient populations were analyzed: (a) all patients with an LVEF ≤ 35% before TAVI (overall study population), and (b) a subpopulation of patients with documented echocardiography and persistent LV dysfunction (LVEF ≤ 35%) as determined at the 3-month follow-up after TAVI. Patients of both groups were further divided into those with or without an ICD at discharge. Those patients without an ICD at discharge who reported an ICD implantation during the observation period were censored as alive at the timepoint of the implantation. Type and dosage of heart failure medication (% of recommended target dose) at the time of hospital discharge after TAVI were assessed. For the purpose of this study, patients taking angiotensin receptor neprilysin inhibitors (ARNI) were classified as taking a renin-angiotensin system inhibitor. Follow-up examinations were scheduled at 3 months (ambulatory visits) followed by annual follow-up telephone calls. Follow-up data were obtained by outpatient visits, telephone interviews, or by medical reports from referring physicians. The study was conducted in accordance with the Declaration of Helsinki, and the protocol was approved by the Ethics Committee of the University of Giessen. Due to the retrospective nature of this study a waiver of written informed consent was issued by the ethics committee.

### 2.2. Outcome Variables

The primary endpoint was death from any cause within three years post-intervention. Patients who were alive after the three-year follow-up were censored as alive after three years. Secondary endpoints were the incidences of specific causes of CV death according to the Valve Academic Research Consortium-2 consensus document [8].

### 2.3. Echocardiographic Measurements

Echocardiographic exams were scheduled before TAVI and at the 3-month follow-up. EF was estimated visually. The stroke volume was determined at the LV outflow tract by multiplying the cross-sectional area by the systolic velocity integral. Aortic valve area was calculated according to the continuity equation. In patients with insufficient visualization, transesophageal echocardiography was performed to measure aortic valve area by planimetry. Low-flow, low-gradient AS was diagnosed according to current guidelines [9].

### 2.4. Statistical Analysis

Continuous data were tested for normal distribution and are reported as median and interquartile range (IQR). Continuous values were compared by the Mann-Whitney Kruskal-Wallis test and categorical variables by the χ^2^ test. Survival curves were constructed using Kaplan-Meier estimates and were compared by the log-rank test. Univariate Cox regression analysis was performed to test the impact of baseline variables on mortality; those parameters with significant univariable impact (*p* < 0.1) entered a multivariable analysis. All statistical analyses were performed using the SPSS statistical package version 26 (IBM Corp., Armonk, NY, USA).

## 3. Results

**Figure 1:**
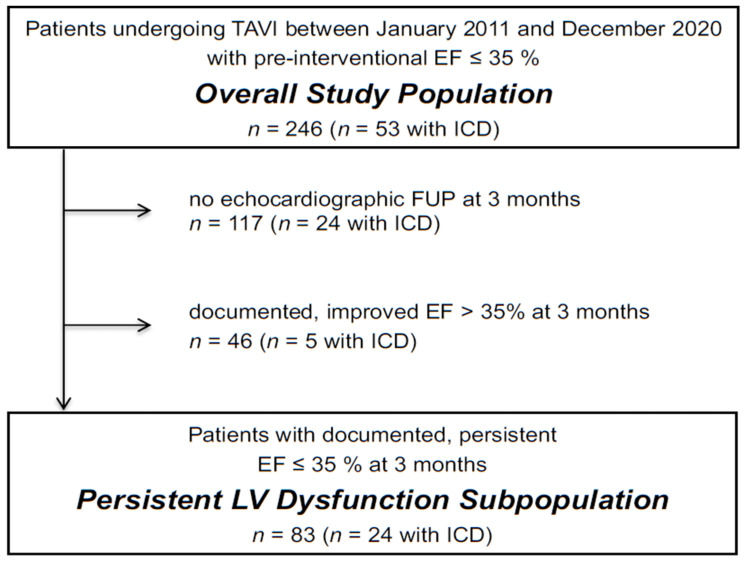
Flowchart illustrating the makeup of the study populations. Abbreviations: EF, ejection fraction; FUP, follow-up; ICD, implantable cardioverter defibrillator.

We identified 246 patients with an LVEF ≤ 35% at baseline (overall study population). Echocardiography at the first follow-up after a median of 91 (66–102) days was performed in 129 patients of the overall study population. Of those, 83 patients had a consistently reduced LVEF ≤ 35% (persistent LV dysfunction subpopulation), whereas 46 patients had an improved LVEF > 35 % (Figure 1 and Table 1).

Patients of the overall study population with an ICD were more often male and were slightly younger than those without an ICD; they had a higher prevalence of obstructive lung disease but did not differ with respect to the prevalence of manifest cardiovascular disease or severity of clinical symptoms. Calculated perioperative risk tended to be higher in patients with an ICD but the difference was not significant. Biventricular pacing was almost exclusively a characteristic of patients with an ICD. The majority of patients included fulfilled the criteria for the diagnosis of low-flow, low-gradient AS. The use of balloon-expandable prosthesis and the TAVI device success rates were not different between groups. More than 86% of all patients were on beta-blockers and more than 77% were on renin-angiotensin system inhibitors at the timepoint of hospital discharge, without significant differences between groups (Table 2).

More patients with an ICD took mineralocorticoid receptor antagonists, but the number of patients taking ≥50% of the target dose was similar. Due to our registry’s inclusion period of 10 years, less than 8% of all patients were on the relatively modern ARNI therapy. Among patients with persistent LV dysfunction, baseline characteristics were well matched between those with or without an ICD with the exception that those with an ICD were several years younger. No differences in the prevalence of baseline cardiovascular diseases and no differences in calculated risk, TAVI success rates, or heart failure medication at discharge were detected.

**Figure 2:**
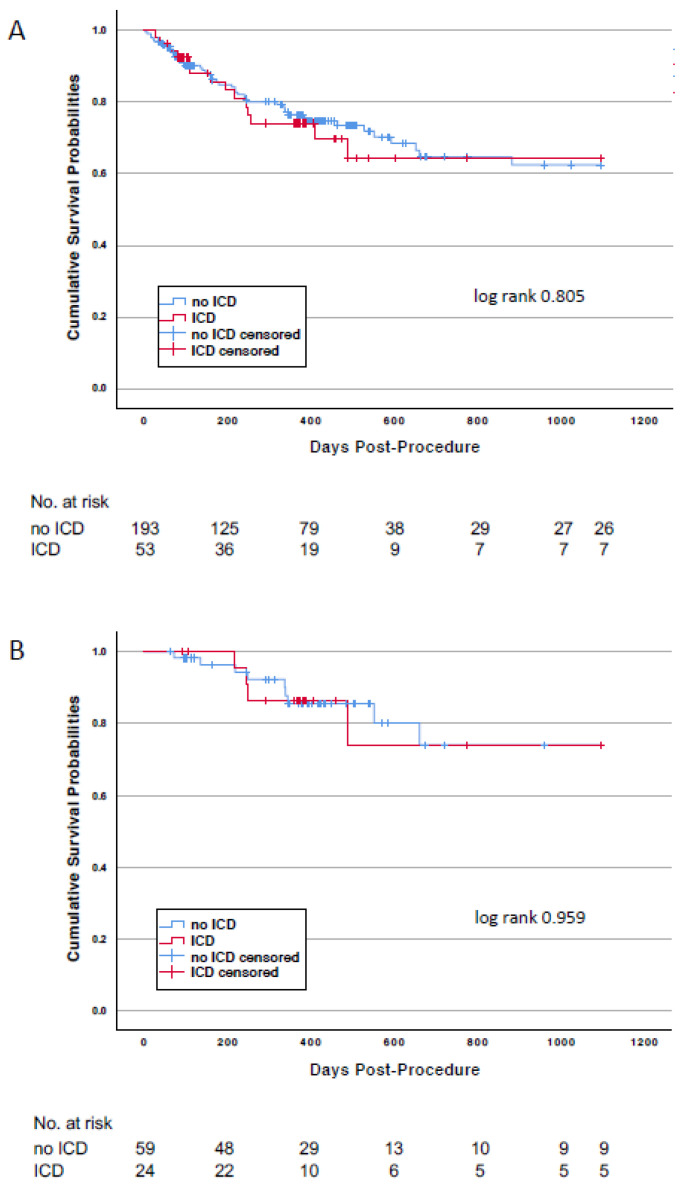
Kaplan-Meier survival curves for patients with or without an ICD at discharge after TAVI. Panel (**A**): Patients of the overall study population. Panel (**B**): Patients of the persistent LV dysfunction subpopulation.

The median follow-up time was 370 (105–500) days. During the follow-up time, four patients were implanted with an ICD and were censored as alive at this timepoint. Mortality rates in the overall study population at 3 years were 26.4% for patients with an ICD and 24.4% (*p* = 0.758) for patients without an ICD, respectively (Figure 2 and Table 3). Cardiovascular death rates between the two groups were similar. In both groups, most deaths were caused by the worsening of HF. Sudden cardiac death was observed in none of the patients with an ICD and in 3 patients (8.8%) without an ICD.

Mortality in the study subpopulation with persistent LV dysfunction was lower than that of the overall study population (Table 3) due to the fact that for this landmark-like analysis only patients surviving the first follow-up visit at 3 months qualified for inclusion. Mortality at 3 years was 16.7% in patients with an ICD and 15.3% in those without (*p* = 0.872). Again, worsening of HF accounted for more than 50% of all cardiovascular deaths in both groups. No death was qualified as sudden cardiac death.

Survival was further analyzed in two groups of the overall study population. Patients with previous myocardial infarction displayed mortality rates of 26.7% in patients with an ICD and 22.2% in those without (log-rank *p* = 0.952). Patients with low-flow, low-gradient AS and extraordinarily high cardiovascular risk had mortality rates of 30.8% and 28.1% for those with or without an ICD, respectively (log-rank *p* = 0.778).

**Figure 3:**
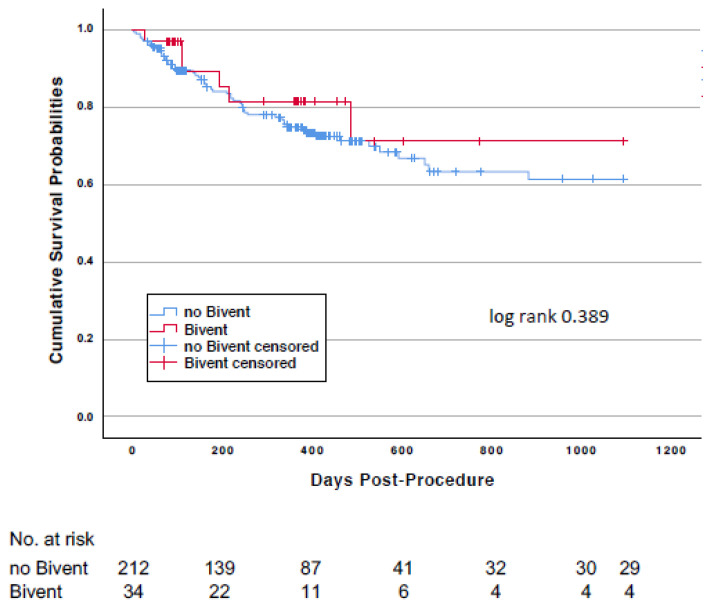
Kaplan-Meier survival curves comparing patients with and without biventricular pacing.

The effect of biventricular pacing (*n* = 34) on survival was analyzed for the overall study population. Mortality at 3 years was observed in 6 (17.6%) vs. 55 (25.9%) (log-rank *p* = 0.389) patients with or without biventricular pacing (Figure 3), and cardiovascular mortality occurred in 5 (14.7%) vs. 38 (17.9%) (*p* = 0.646), respectively.

Variables with potential impact on mortality were tested in univariable and multivariable analyses to assess their prognostic value (Table 4). Renal function, a history of atrial fibrillation, the EuroScore II, and a diagnosis of low-flow, low-gradient AS emerged as predictors of mortality, and the glomerular filtration rate as well as the EuroScore were identified as independent predictors. The presence of an ICD or biventricular pacing had no impact on mortality.

Patient characteristics of the 117 patients of the overall study population without an echocardiographic follow-up examination (*n* = 117) are given in Supplementary Table S1. In general, baseline characteristics of patients with or without ICD were similar to those of the overall study population. The 117 patients without echocardiographic follow-up had slightly higher ESII scores and higher mortality rates compared with the patients of the overall study population. However, 3-year mortality was not different between patients with (10/24; 41.7%) and without (35/93; 37.6%) an ICD (*p* = 0.717).

Patients of the overall study population with an improved LVEF at follow-up examination (*n* = 46) had a significantly lower prevalence of CAD and of prior myocardial infarction and a significantly lower EuroScore II compared with those having a continuously depressed LVEF at three months follow-up (Supplementary Table S1). During the 3-year follow-up, 0/5 patient with an ICD and 3/41 (7.3%) without an ICD died (*p* = 0.579).

## 4. Discussion

This retrospective study examined the effect of an ICD during a longer-term follow-up period post-TAVI in patients with reduced EF who can be considered as potential candidates for ICD therapy. During the observation period, patients with an ICD did not have improved survival compared with that of patients without. Given the uncertainty of diagnosing HF in patients with severe AS and overlapping clinical, laboratory, and echocardiographic parameters, and considering the fact that some of these patients’ LV function would recover after correction of afterload, we also analyzed the effect of an ICD in patients with continuously depressed LV function three months after TAVI. Again, these patients did not benefit from an ICD therapy. These results were also confirmed in patients with previous myocardial infarction and in those with low-flow, low-gradient AS at highest risk. Interestingly, worsening of HF was the predominant cause of all cardiovascular deaths, whereas only a few of those patients without an ICD were classified as having died from sudden cardiac death. Accordingly, biventricular pacing appeared to be a potential therapeutic option for these patients, although the prognostic effect of this therapy was not significant. Taken together, our data do not support initiating ICD therapy in these patients post-TAVI as a part of regular clinical practice.

While the reported prevalence of HF of ischemic, idiopathic, or valvular etiology in patients with AS undergoing TAVI varies widely—from 10–40% based on the definition of HF [10]—there is unanimous agreement on the negative prognostic impact of HF in patients with AS after TAVI. [2,3,4,11,12]. Therefore, one can speculate that ICD implantation in addition to medical HF therapy could save lives in these patients. However, cardiovascular deaths reported by these studies are related rather to hemodynamic consequences of HF than to arrhythmic adverse events in the post-TAVI period. Indeed, data on the burden of ventricular arrhythmias following TAVI are very rare. One single study using 24-h Holter monitoring performed 1-year post-TAVI reported electrocardiographic data in a study cohort of 146 patients: the rate of ventricular tachycardia was only 2% and it was non-sustained [13]. Thus, it appears that ventricular tachycardias do not present a substantial trigger for adverse events in this patient population, an observation supporting the conclusion of our study. It would be helpful to classify the etiology of HF, given that the beneficial effect of an ICD may depend on the ischemic [14] or non-ischemic [15] origin of the ventricular arrhythmias. However, we believe that such an attempt is not feasible in patients with concomitant severe AS, as the influence of the “valvular heart disease” will always be an unresolved issue that prevents a clear classification of etiologies. For the same reasons, we are skeptical about the usefulness of classical HF scores to estimate risk and to predict outcomes in our patients with or without ICD. In fact, the well-established Seattle Heart Failure score [16] has not been evaluated in patients with concomitant severe AS and was shown to perform suboptimally in older patients [17]. Furthermore, the risk score derived from older patients of the SENIORS trial [18] does not take into account the effects of any device therapy. Therefore, one has to be cautious in comparing outcomes of our study with those of classical HF trials.

Several considerations could explain the neutral outcome of our study. First, recommended therapies have often been validated in younger patient populations but are increasingly applied to elderly patient populations where effects have not yet been studied. For instance, the mean ages of the patients investigated in the Multicenter Automatic Defibrillator Implantation II (MADIT II) [14] and Sudden Cardiac Death in Heart Failure (SCD-HeFT) [19] trials, which led to the recommendation of ICD therapy for primary prophylaxis [1], were 64 and 60 years, respectively, and the prevalence of concomitant manifest cardiovascular disease in those patients was rather low. Thus, it remains a matter of debate whether considerably older patients like those treated by TAVI may also profit from ICD therapy [20,21,22], as data on ICD use in the increasing population of the oldest elderly patients are simply lacking. Second, typical patients undergoing TAVI may be too sick to profit from ICD therapy, even when life expectancy should be considered by referring physicians to be more than one year in all patients undergoing TAVI. In one study investigating survival times after ICD implantation in octogenarians, median survival was cut from 4.7 years to 19 months if patients had a severely reduced EF ≤ 30% and an estimated glomerular filtration rate < 60 mL/min [23], corroborating the fact that comorbidities lead to a severe reduction in survival independently of ICD therapy [24]. As survival curves after TAVI mostly overlap before this timespan between patients with an ICD and those without, it is reasonable to expect that no effect of ICD therapy may be observed in this patient population. Third, it remains unclear how effectively ICD therapy in our study population prevented sudden cardiac death. The relatively small number of such events in our patients without an ICD may be due to the fact that sudden cardiac death does not usually manifest with specific symptoms and may be wrongly diagnosed as worsening HF or as death of an unknown cause, explaining the rather high number of this latter classification in our study.

## 5. Limitations of the Study

This study has several limitations that should be considered. It is a retrospective analysis with all the limitations inherent to such a study design. Echocardiographic measurements were made by different operators without a centralized core lab, and EF was estimated visually. There was no EF reported after 3 months; therefore, potential improvements after this timepoint may have been missed. Indication for a prior ICD therapy in patients referred for TAVI could not be ascertained in most patients, which may have led to a selection bias. Finally, follow-up information was partly dependent on reports from third parties or information from patients’ relatives, which could have led to underreporting or misclassification of events.

## 6. Conclusions

Taken together, the results of our study do not provide evidence for any benefits of ICD therapy in patients with reduced EF undergoing TAVI on a regular basis. Rather, these findings support the skepticism surrounding the application of such a therapy in very old patients with a high burden of concomitant disease.

## Figures and Tables

**Table 1:** Patient Characteristics.

	Overall Study Population			Persistent LV Dysfunction Subopulation		
	no ICD	ICD	*p*	no ICD	ICD	*p*
	*n* = 193	*n* = 53		*n* = 59	*n* = 24	
**Demographic data**						
Female	56 (29.0)	8 (15.1)	0.041	17 (28.8)	6 (25.0)	0.725
Age, year	81 (77–85)	77 (73–80)	<0.001	80 (77–83)	77 (71–79)	0.004
BMI, kg/m^2^	25.9 (23.8–29.1)	27.7 (24.4–30.1)	0.074	26.9 (24.1–30.6)	28.0 (23.4–29.5)	0.984
Diabetes mellitus	84 (43.5)	30 (56.6)	0.091	28 (47.5)	13 (54.2)	0.579
GFR, mL/min/1.73 m^2^	56 (42–72)	52 (36–68)	0.225	55 (42–71)	52 (32–67)	0.155
Anemia	74 (38.3)	15 (28.3)	0.178	23 (39.0)	6 (25.0)	0.226
COPD	23 (11.9)	15 (28.3)	0.003	8 (13.6)	4 (16.7)	0.715
**Cardiovascular disease**						
CAD	132 (68.4)	36 (67.9)	0.948	41 (69.5)	17 (70.8)	0.904
Prior MI	45 (23.3)	15 (28.3)	0.454	18 (30.5)	7 (29.2)	0.904
History of atrial fibrillation	91 (47.2)	30 (56.6)	0.223	25 (42.4)	13 (54.2)	0.328
Prior stroke	21 (10.9)	3 (5.7)	0.257	3 (5.1)	1 (4.2)	0.859
Peripheral artery disease	32 (16.6)	12 (22.6)	0.308	10 (16.9)	6 (25.0)	0.399
Prior cardiac decompensation	114 (59.1)	31 (58.5)	0.940	34 (57.6)	11 (45.8)	0.328
NYHA class III / IV	173 (89.6)	46 (86.8)	0.557	52 (88.1)	21 (87.5)	0.936
EuroScore II, %	6.6 (4.6–11.1)	7.8 (5.4–12.5)	0.227	6.3 (4.6–11.7)	6.9 (4.8–14.9)	0.382
**Echocardiographic data**						
Ejection fraction, %	30 (25–33)	29 (25–30)	0.164	28 (25–30)	25 (20–30)	0.578
LFLG-AS	121/174 (69.5)	39/48 (81.3)	0.109	41/51 (80.4)	16/20 (80.0)	0.970
≥moderate MR or TR	72 (37.3)	18 (34.0)	0.654	19 (32.2)	9 (37.5)	0.644
**Device therapy**						
Pacemaker at discharge	32 (16.6)	0	0.001	4 (6.8)	0	0.191
Biventricular pacing	1 (0.5)	33 (62.3)	<0.001	0	17 (70.8)	<0.001
**Procedural data**						
Balloon-expandable valve	92 (47.7)	31 (58.5)	0.163	26 (44.1)	13 (54.2)	0.403
Device success	158 (81.9)	44 (83.0)	0.846	50 (84.7)	22 (91.7)	0.399
≥moderate residual aortic regurgitation	2 (1.1)	2 (3.8)	0.201	0	1 (4.2)	0.121

Data shown as number (%) or median (interquartile range). Abbreviations: ICD = implantable cardioverter defibrillator; BMI = body mass index; GFR = glomerular filtration rate (estimated); COPD = chronic obstructive pulmonary disease; NYHA = New York Heart Association; CAD = coronary artery disease; CABG = coronary artery bypass grafting; LFLG-AS = low-flow, low-gradient aortic stenosis; MI = myocardial infarction; MPG = mean pressure gradient; MR = mitral regurgitation; TR = tricuspid regurgitation.

**Table 2:** Medication at hospital discharge.

	Overall Study Population			Persistent LV Dysfunction Subpopulation		
	no ICD	ICD	*p*	no ICD	ICD	*p*
	*n* = 193	*n* = 53		*n* = 59	*n* = 24	
Beta-blockers	165 (85.6)	49 (92.5)	0.182	57 (96.6)	22 (91.7)	0.340
≥50% target dose	84 (43.5)	21 (39.6)	0.611	28 (47.5)	7 (29.2)	0.126
RAS blockers	150 (77.7)	41 (77.4)	0.955	46 (78.0)	21 (87.5)	0.318
≥50% target dose	56 (29.0)	16 (30.2)	0.868	17 (28.8)	10 (41.7)	0.257
MR antagonists	112 (58.0)	40 (75.5)	0.021	40 (67.8)	16 (66.7)	0.921
≥50% target dose	103 (53.4)	34 (64.2)	0.162	37 (62.7)	14 (58.3)	0.710
ARNI	9 (4.7)	4 (7.5)	0.406	6 (10.2)	2 (8.3)	0.797
≥50% target dose	4 (2.1)	0	0.291	2 (3.4)	0	0.361

Data are numbers (%). Abbreviations: LV = left ventricular; ICD = implantable cardioverter defibrillator; RAS = renin-angiotensin system; MR = mineralocorticoid receptor; ARNI = angiotensin receptor-neprilysin inhibitors.

**Table 3:** Primary and secondary outcomes.

	Overall Study Population			Persistent LV Dysfunction Subpopulation		
	no ICD	ICD	*p*	no ICD	ICD	*p*
	*n* = 193	*n* = 53		*n* = 59	*n* = 24	
Primary Outcome						
All-cause mortality at 3 years	47 (24.4)	14 (26.4)	0.758	9 (15.3)	4 (16.7)	0.872
Secondary Outcomes						
CV death at 3 years	34 (17.6)	9 (17.0)	0.914	7 (11.9)	4 (16.7)	0.559
myocardial infarction	0	1 (11.1)		0	1 (25.0)	
worsening HF	8 (23.5)	4 (44.4)		5 (71.4)	2 (50.0)	
neurological events	2 (5.9)	1 (11.1)		0	0	
pulmonary embolism	1 (2.9)	0		0	0	
other vascular disease	1 (2.9)	0		0	0	
procedure related	1 (2.9)	0		0	0	
sudden cardiac death	3 (8.8)	0		0	0	
death of unknown cause	18 (52.9)	3 (33.3)		2 (28.6)	1 (25.0)	

Incidence of mortalities is shown as number (%). Incidence of specific causes of CV mortality is shown as number and percentage of CV deaths. Abbreviations: CV = cardiovascular; ICD = implantable cardioverter defibrillator; LV = left ventricular; HF = heart failure.

**Table 4:** Univariable and multivariable Cox regression analysis for prediction of mortality.

Variable	HR	CI 95%		*p*	HR	CI 95%		*p*
		Lower	Upper			Lower	Upper	
Age, years	1.012	0.977	1.049	0.503				
Sex (female/male)	1.262	0.695	2.293	0.445				
BMI, kg/m^2^	0.987	0.935	1.042	0.638				
GFR, ml/min/1.73 m^2^	0.983	0.972	0.995	0.004	0.989	0.976	1.003	0.117
Diabetes (no/yes)	0.933	0.564	1.544	0.787				
CAD (no/yes)	1.695	0.933	3.082	0.083	1.089	0.565	2.099	0.8
History of AF (no/yes)	1.8	1.067	3.036	0.028	1.799	1.022	3.164	0.042
Prior decomp (no/yes)	1.211	0.721	2.033	0.469				
EuroScore II, %	1.063	1.025	1.101	0.001	1.055	1.013	1.099	0.01
Pacemaker (no/yes)	0.949	0.451	1.996	0.889				
Bivent (no/yes)	0.692	0.298	1.607	0.391				
ICD (no/yes)	1.078	0.593	1.959	0.805				
Ejection fraction, %	0.975	0.934	1.017	0.232				
LFLG-AS (no/yes)	2.154	1.054	4.403	0.035	1.678	0.798	3.527	0.172
≥moderate MR or TR (no/yes)	1.051	0.626	1.765	0.85				
Balloon-expandable valve (no/yes)	0.965	0.584	1.594	0.888				
≥moderate AR post-TAVI (no/yes)	2.246	0.547	9.219	0.261				
HF medication (no/yes)	0.686	0.414	1.138	0.144				

AF = atrial fibrillation; CAD, coronary artery disease; CI, confidence interval; GFR, glomerular filtration rate; HR, hazard ratio; decomp = cardiac decompensation; ICD = implantable cardioverter defibrillator; Bivent = biventricular pacing; LFLG-AS = low-flow low-gradient aortic stenosis; HF medication = RAS antagonist+beta-blocker+mineralocorticoid receptor antagonist, MR = mitral regurgitation; TR = tricuspid regurgitation.

## Data Availability

Data cannot be made publicly available for ethical or legal reasons, e.g., public availability would compromise patient confidentiality or participant privacy. Data are available from the Kerckhoff Institutional Data Access for researchers who meet the criteria for access to confidential data. Any requests for data access may be sent to the administration of the Kerckhoff Heart Center via email at info@kerckhoff-klinik.de or by contacting: Kerckhoff-Klinik GmbH, Geschäftsführung, Benekestrasse 2-8, 61231 Bad Nauheim.

## Supplementary Materials

The following are available online at https://www.mdpi.com/article/10.3390/jcm10132929/s1, Table S1: Patients with no echocardiographic FUP at 3 months.
