# Supplementary material for: Long-Term Survival in Patients with or without Implantable Cardioverter Defibrillator after Transcatheter Aortic Valve Implantation
Source: J Clin Med. 2021 Jun 30;10(13):2929. doi: 10.3390/jcm10132929 (PMC8268788; doi:10.3390/jcm10132929)
Supplement: Supplementary file 1 [file jcm-10-02929-s001.zip › jcm-1236411-supplementary.pdf]

Supplementary Table S1. Patients with no echocardiographic FUP at 3 months.

	no ICD	ICD	*p*
	n = 93	*n* = 24	
**Demographic data**			
Female	25 (26.9)	1 (4.2)	0.017
Age, y	82 (78–87)	78 (74–81)	0.003
BMI, kg/m²	25.3 (23.6–27.7)	27.3 (25.2–32.0)	0.026
Diabetes mellitus	39 (41.9)	14 (58.3)	0.150
GFR, mL/min/1.73 m²	52 (35–71)	55 (36–71)	0.821
Anemia	35 (37.6)	7 (29.2)	0.441
COPD	9 (9.7)	10 (41.7)	<0.001
**Cardiovascular disease**			
CAD	70 (75.3)	17 (70.8)	0.657
Prior MI	22 (23.7)	7 (29.2)	0.577
History of atrial fibrillation	46 (49.5)	15 (62.5)	0.254
Prior stroke	15 (16.1)	1 (4.2)	0.128
Peripheral artery disease	17 (18.3)	6 (25.0)	0.460
Prior cardiac decompensation	53 (57.0)	17 (70.8)	0.217
NYHA class III / IV	84 (90.3)	20 (83.3)	0.331
EuroScore II, %	7.3 (5.3–12.7)	8.4 (4.2–11.2)	0.855
**Echocardiographic data**			
Ejection fraction, %	30 (25–30)	30 (25–30)	0.560
LFLG-AS	59/85 (69.4)	20/23 (87.0)	0.092
≥ moderate MR or TR	21 (22.6)	8 (33.3)	0.277
**Device therapy**			
Pacemaker at discharge	21 (22.6)	0	0.010
Biventricular pacing	1 (1.1)	14 (58.3)	<0.001
**Procedural data**			
Balloon-expandable valve	47 (50.5)	15 (62.5)	0.295
Device success	74 (79.6)	18 (75.0)	0.626
≥ moderate residual aortic regurgitation	1/82 (1.2)	1/24 (4.2)	0.351
